# Proteomic Analysis of *Corpora Amylacea* Extracted From *Post‐mortem* Brain of MAiD‐end‐of‐life Sporadic ALS Patients

**DOI:** 10.1002/brb3.71486

**Published:** 2026-05-24

**Authors:** Alexandre Paquet, Lydia Touzel‐Deschênes, Vincent Roy, Stephan Saikali, Nicolas Dupré, François Gros‐Louis

**Affiliations:** ^1^ Department of Surgery, Faculty of Medicine Université Laval Quebec City QC Canada; ^2^ Regenerative Medicine Research Program CHU De Québec‐Université Laval Research Center Québec QC Canada; ^3^ Department of Medical Biology, Division of Anatomic Pathology and Neuropathology CHU De Québec Quebec City QC Canada; ^4^ Department of Medicine, Faculty of Medicine Laval University Quebec City QC Canada; ^5^ Neuroscience Research Program CHU De Québec‐Université Laval Research Center Québec QC Canada

**Keywords:** Corpora amylacea, ALS, mass spectrometry, neurodegeneration, biomarkers

## Abstract

**Purpose:**

*Corpora amylacea* (CA) are starch‐like inclusions that accumulate in the central nervous system (CNS) with aging and are enriched in neurodegenerative conditions, including amyotrophic lateral sclerosis (ALS). Although often regarded as waste reservoirs, their cellular origins, molecular composition, and pathological significance remain poorly understood.

**Methods:**

Here, we performed an unbiased proteomic analysis of purified CAs isolated from *post‐mortem* brains of sporadic ALS patients and controls.

**Findings:**

In‐depth mass spectrometry identified 4,470 proteins, of which 658 were quantified, revealing distinct ALS‐specific proteomic signatures. Enriched proteins included markers of cytoskeletal remodeling, mitochondrial dysfunction, and proteostasis disruption, as well as known ALS‐associated proteins such as TDP‐43 and neurofilament proteins. These findings demonstrate that CAs serve as reservoirs of dysfunctional, disease‐relevant proteins and capture key pathological processes in ALS.

**Conclusion:**

By applying an unbiased proteomic approach to purified CAs, this study provides the first comprehensive map of their protein content in ALS, supporting their potential as biomarker sources and as a source of mechanistic insights into neurodegeneration.

**Significance:**

Unbiased analyses of CAs in the context of ALS have yet to be undertaken. This study provides the first proteomic profiling of purified CAs, isolated from ALS patient brains using biochemical methods, revealing that CAs harbor disease‐relevant proteins implicated in sporadic ALS. By demonstrating that CAs act as reservoirs of dysfunctional proteins related to metabolism, cytoskeletal organization, and proteostasis, our findings highlight their potential as a novel source of ALS‐specific mechanistic insight into disease pathology.

## Graphical abstract

*Corpora amylacea* extracted from the brain of ALS patients carry a specific proteomic signature. Created in BioRender. Paquet, A. (2025) https://BioRender.com/x6dh8e0

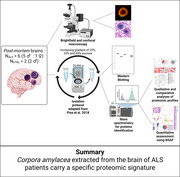

## 1 Introduction

Amyotrophic Lateral Sclerosis (ALS) is a progressive neurodegenerative disease that affects motor neurons in the brain and spinal cord, leading to loss of muscle control. Over time, individuals with ALS lose the ability to speak, eat, move, and breathe independently, with a severe prognosis of 3–5 years post‐diagnosis (Masrori and Van Damme 2020). Neuronal loss in ALS primarily results from the accumulation of pathological, phosphorylated forms of TDP‐43, which disrupt essential cellular processes and drive neurodegeneration. Despite intensive research, there are currently no curative therapies, and disease‐modifying treatments remain limited. A major obstacle in ALS research and clinical management is the absence of reliable biomarkers that can aid in *post‐mortem* disease stratification, which is critical for understanding complex neurodegenerative diseases, as well as for early diagnosis, tracking disease progression, and serving as outcome measures for therapeutic trials. To address this unmet need, new biological reservoirs beyond conventional biofluids, such as cerebrospinal fluid (CSF) and blood, must be explored. Ideally, such sources would capture disease‐specific pathological processes and could be studied in a reproducible, unbiased manner. *Post‐mortem* brain tissue remains a valuable resource for understanding ALS pathophysiology, and its structures may provide unique insights into disease‐associated molecular signatures. Among these, *corpora amylacea* (CAs) represent a particularly intriguing candidate. First reported in the central nervous system (CNS) by Purkinje in 1839 and later described by Rudolf Virchow (Riba et al. 2023), CAs have long been recognized but remain enigmatic.

CAs are spherical and granular starch‐like bodies found in the CNS, particularly in the brain (Augé et al. 2019), and in other nervous tissues like the retina, olfactory tract and optic nerves (Avendano et al. 1980). These bodies mainly comprise polyglucans, lipids, and proteins, and vary in composition, size, and localization (Riba et al. 2021, Riba et al. 2019, Riba et al. 2022). Although their function remains debated, they are thought to act as cellular waste containers, sequestering damaged proteins, lipids, and other metabolic by‐products. CAs accumulation is associated with aging (Rohn 2015). In addition, the augmented abundance of CAs in the brain of patients has also been linked to Alzheimer's disease (Zhan et al. 2021) and other neurodegenerative pathologies including ALS and frontotemporal lobar degeneration (FTLD) (Atsumi 1981, Pisa et al. 2018, Pisa et al. 2016, Alsina et al. 2024, Paré et al. 2018). Their molecular composition has been only partially characterized, and very little is known about their potential role and relevance in ALS. A connection that could be driven by the accumulation of pathological proteins such as TDP‐43, TAU, FUS, and misfolded SOD1, common hallmarks of both ALS and FTLD (Alsina et al. 2024, Paré et al. 2018).

Although the exact functions and mechanisms of accumulation of these bodies within the CNS remain unclear, it has been proposed that CAs may accumulate as end‐products of various cellular processes or as artifacts, partly reflecting methodological limitations. Among these, glycogen metabolism defects, protein aggregates from lymphatic or hematogenous sources, accumulations of breakdown products from neurons and oligodendrocytes, remnants of degenerating neurons, and conglomerations of interacting proteins from degenerating neurons and blood elements have been mentioned (Riba et al. 2021, Riba et al. 2019, Riba et al. 2022).

Nearly two centuries after their discovery, their exact origin, accumulation and function continue to be debated, with hypotheses ranging from protective waste reservoirs of glial origin to possible *post‐mortem* artifacts or even microbial remnants (Riba et al. 2021). The presence of waste elements is a recurring theme, suggesting that CAs may trap and sequester potentially harmful substances or serve as a resistance mechanism for cleaning the CNS, which remains the current consensus to this day (Riba et al. 2021, Cavanagh, Apr 1999, Augé et al. 2018, Augé et al. 2018, Auge et al. 2017). Recent studies reinforced this view by showing that CAs express neoepitopes, abnormal protein forms absent in normal tissues, that enable their recognition and phagocytosis by macrophages (Auge et al. 2017). These findings suggest that CAs are not random by‐products but components of a structured waste management system, accumulating in an organized manner that often follows a gradient around perivascular regions of the brain (Riba et al. 2019). Furthermore, proteins account for about 4–5% of the CA's dry weight (Pirici and Margaritescu 2014).

In this study, we report a method to isolate and purify CAs from *post‐mortem* brains collected from sporadic ALS (sALS) patients who received medical assistance in death (MAiD), and to demonstrate the feasibility of performing unbiased proteomic profiling.

We show that CAs harbour distinct protein signatures associated with ALS pathology. Our findings open the way to future studies investigating CAs to better understand disease pathogenesis and as biomarker sources across neurodegenerative diseases such as ALS.

## 2 Materials and Methods

### 2.1 *Post‐mortem* Brains Biopsies and Ethics

This study received approval from the national ethical review boards in Canada (Ethical Research Board of the CHU de Québec, Protocol number: 2012‐1286 (PEJ‐235), contact: gurecherche@chuq.qc.ca). Informed consent was obtained from all participants who participated in the Canadian MAiD program. Human autopsies were conducted according to established protocols by the staff of Enfant‐Jesus Hospital Pathology Department. *Post‐mortem* brain tissues were collected from autopsy material, including six sALS cases and two non‐ALS controls, who were individuals diagnosed with autosomal recessive spastic ataxia of Charlevoix‐Saguenay (ARSACS), primary angiitis of the central nervous system (PACNS) (Supplementary Table 1). The *post‐mortem* interval was inferior to 24 h following the MAiD procedure. After extraction, brain tissues were rapidly transferred to the neuropathological service of the CHU de Québec in isotonic medium (Dulbecco's Modified of Eagle Media; Invitrogen, Burlington, ON, Canada) before processing.

### 2.2 CAs Identification by Histology and Immunofluorescence

Brain samples were initially fixed in 4% paraformaldehyde (Electron Microscopy Sciences, Hatfield, PA, USA), embedded in paraffin, and cut into 10‐µm‐thick cross‐sections on glass slides. Brain tissues on glass slides were stained with hematoxylin and eosin for histology analyses following the standard procedure (Paré et al. 2015).

For immunofluorescence analyses, paraffin was removed in a 100% toluene bath for 5 min, followed by a 100% ethanol bath for 5 min. These tissues underwent antigenic unmasking in a 10 mM citrate buffer, pH 6.0, at 95°C for 15 min and were blocked with 5% goat serum (Invitrogen, Waltham, Massachusetts, USA) and 0.3% Triton X‐100 (Bio‐Rad Laboratories Inc., Hercules, CA, USA) in phosphate‐buffered saline (PBS) for 1 h at room temperature. Specific primary antibodies against p62 (SQSTM; 1:200; ab56416; Abcam, Cambridge, UK) and glycogen synthase (GYS; 1:200; S3886; Cell Signaling Technology, Danvers, MA, USA) were incubated on the tissues for 16 h at 4°C. After washing, the tissues were then incubated for 1 h at room temperature with Alexa Fluor 594‐labeled goat anti‐rabbit IgG (H+L) (1:500; A21131; Invitrogen) for p62 and Alexa Fluor 488‐labeled goat anti‐mouse IgG (1:500; A11012; Invitrogen) for GYS. Finally, tissues were incubated with 0.1% Sudan Black B (MilliporeSigma, Burlington, MA, USA; S0593) at room temperature for 30 min and mounted with fluoromont‐G with DAPI (Invitrogen; 00‐4959‐52). The immunostained tissues were visualized using an LSI 700 confocal microscope with a Zeiss Axio Imager (Carl Zeiss Microscopy, Jena, Germany).

### 2.3 CAs Isolation

The following protocol is adapted from Pisa et al., 2018^11^. CAs were extracted from *post‐mortem* brain samples of six ALS patients (ALS) and two non‐ALS controls (CTRL). The extraction protocol consists of a succession of centrifugations in increasing sucrose gradients of 25%, 35% and 45% with various treatments with DNAse, RNAse and sodium deoxycholate.

350 mg samples of each brain's right exterior hemisphere's prefrontal cortex were homogenized in 300 µL of PBS with CaCl_2_ at 0.131 g/L (PBS‐Ca). 1.5 mL of PBS‐Ca added with sucrose was added to achieve a final concentration of 25% in 1.8 mL, and the samples were then centrifuged at 20,000 x *g* for 5 min. The pellets were resuspended in 1.8 mL of 25% sucrose in PBS‐Ca and centrifuged again at 20,000 x *g* for 10 min. The pellet was resuspended in 300 µL of PBS‐Ca containing 1% sodium deoxycholate (Bio Basic Inc., Markham, ON, Canada), then 1.5 mL of PBS‐Ca with sucrose was added to a final concentration of 25%, and the tubes were centrifuged at 20,000 x *g* for 10 min. The pellet was treated at 37°C for 5 min with 1,000 IU of RNAse T1 (Invitrogen) and 15 IU of DNAse (MilliporeSigma) in 300 µL of PBS‐Ca. The volume was adjusted to 1.8 mL with 25% sucrose and centrifuged at 20,000 x *g* for 10 min. The pellet was resuspended in 300 µL of 1.5% sodium deoxycholate in PBS‐Ca, mixed with 1.5 mL of 35% sucrose in PBS‐Ca, and centrifuged at 20,000 x *g* for 10 min. The pellet was again treated with 1,000 IU RNAse T1 and 15 IU DNAse at 37°C for 5 min, followed by adding 1.5 mL PBS‐Ca containing 35% sucrose and centrifugation at 20,000 x *g* for 10 min. This RNAse T1 and DNAse treatment was repeated, then 1.5 mL of 45% sucrose in PBS‐Ca was added, and the samples were centrifuged at 20,000 x *g* for 10 min. Finally, the pellets were resuspended in PBS‐Ca and stored at ‐80°C until use.

### 2.4 Western Blot

Isolated CAs were treated with SUB lysis buffer (MilliporeSigma) overnight at 4°C. The protein concentration of extracted CAs was assessed using Bradford assay with Quick Start Bradford Dye Reagent (Bio‐Rad Laboratories Inc.). 10 mg of proteins per sample were subjected to electrophoresis on a SDS‐PAGE gel with 12% polyacrylamide, then transferred onto a polyvinylidene fluoride blotting membrane (PVFD; Bio‐Rad Laboratories Inc.) and blocked with 5% non‐fat dried milk and 0.05% Tween‐20 (VWR) in tris‐buffered saline (TBS). The membranes were then incubated overnight at 4°C with primary antibodies against p62 (1:1500; ab56416; Biolegend, San Diego, CA, USA), GYS (1:1000; 3886S; Cell Signaling Technology), and TDP‐43 (1:1000; 10782‐2‐AP; Thermo Fisher Scientific, Waltham, MA, USA). Subsequently, membranes were washed in TBS with 0.05% Tween‐20 (VWR) and anti‐rabbit IgG‐HRP (1:5000; Jackson Immunoresearch Laboratories, West Grove, PA, USA) or anti‐mouse IgG‐HRP (1:5000; Jackson Immunoresearch Laboratories) secondary antibodies were applied for one hour at room temperature. Protein detection was carried out using Amersham ECL Western Blotting Detection Reagent (GE Healthcare, Little Chalfont, UK), and bands were imaged with the Fusion Fx7 imager (Vilber Lourmat, France).

### 2.5 General CA Proteins Analysis Workflow

Proteomic identifications were validated by controlling peptide‐level false discovery rates (FDR < 1%). Proteins were retained for downstream analyses if they met a significance threshold (*p* < 0.05) and were supported by at least two unique counted peptides. In total, 4470 proteins were confidently identified. Protein identification was encoded as “presence or absence” binary values (1 or 0) for each sample to assess variability and heterogeneity across samples. PCA was used to summarize global sample structure, and protein detection patterns within the ALS group were visualized using hierarchical clustering heatmaps. To characterize the functional landscape of CAs, the dataset was annotated, and the most enriched Gene Ontology terms (which encompass standardized, hierarchical biological terms and functions) were identified (*p* < 0.05). ALS‐associated proteins were further contextualized through interactome reconstruction using Ingenuity Pathway Analysis software, which incorporates a manually curated database of ALS‐associated genes. Semi‐quantitative analyses were performed on high‐confidence proteins (≥95% confidence, ≥10 peptides) using normalized abundance factors, followed by a limma differential analysis (*p* < 0.05, fold change >1.5) to assess differences between ALS and CTRL samples. Downstream visualizations and functional overrepresentation analyses were conducted in R (*p* < 0.05).

### 2.6 Mass Spectrometry

Proteins were separated on an SDS‐PAGE gel and stained with Coomassie brilliant blue. Gel bands were reduced with 10 mM DTT and alkylated with 55 mM iodoacetamide. Protein digestion was performed using 126 nM of sequencing‐grade modified porcine trypsin (Promega, Madison, WI, USA) for 18 h at 37°C. Digestion products were extracted using 1% formic acid and 2% acetonitrile, followed by 1% formic acid and 50% acetonitrile. Ultimately, the recovered peptide extracts were pooled, vacuum centrifuge dried, and resuspended in 10 µL of 0.1% formic acid for mass spectrometry analysis. Gel electrophoresis, protein digestion, and mass spectrometry analyses were performed by the Proteomics Platform of the CHU de Québec Research Center (Québec, QC, Canada).

Samples were then analyzed by nano liquid chromatography coupled with tandem mass spectrometry (LC‐MS/MS) using a Dionex UltiMate 3000 nanoRSLC chromatography system (Thermo Fisher Scientific) connected to an Orbitrap Fusion mass spectrometer (Thermo Fisher Scientific). Peptides were trapped at 20 µL/min in loading solvent (2% acetonitrile, 0.05% trifluoroacetic acid, TFA) on a 5 mm x 300 µm C18 Pepmap cartridge pre‐column (Thermo Fisher Scientific) for 5 min. Then, the pre‐column was switched online with a Pepmap Acclaim 50 cm x 75 µm internal diameter separation column (Thermo Fisher Scientific). The peptides were eluted with a linear gradient from 5–40% solvent B (A: 0,1% formic acid, B: 80% acetonitrile, 0.1% formic acid) in 200 min, at 300 nL/min. Mass spectra were acquired using data‐dependent acquisition mode with Thermo XCalibur software (version 4.3.73.11; Thermo Fisher Scientific).

Mascot (version 2.8.0; Matrix Science, London, UK) was used to search the UniProt Reference *homo sapiens* database (97134 entries) and a contaminant database. The search assumed the trypsin enzyme digestion and used a fragment ion mass tolerance of 0.60 Da and a parent ion tolerance of 10.0 PPM. Carbamidomethylation of cysteine was specified as a fixed modification, while deamidation of asparagine and glutamine, and oxidation of methionine were specified as variable modifications.

### 2.7 Protein Identification

Scaffold (version Scaffold_5.1.2, Proteome Software Inc., Portland, OR, USA) was used to validate MS/MS‐based peptides and proteins identification. Peptide identification was accepted if a false discovery rate (FDR) of less than 1.0 % was obtained by the Scaffold Local FDR algorithm. Protein identification was significant if it could achieve a *p‐*value of less than 0.05 and contained at least 2 identified peptides within the data. Protein identification probabilities were computed by the Protein Prophet algorithm (Nesvizhskii et al. 2003). Proteins that contained similar peptides and could not be differentiated based on MS/MS analysis alone were grouped to satisfy the principle of parsimony.

### 2.8 Bioinformatics

To assess the CA's protein composition, dataset annotation and gene sets enrichment analysis were performed by the DAVID Functional Annotation online database (Sherman et al. 2022). Only annotations with an enrichment *p*‐value inferior to 0.05 were considered in analyses. The thresholds for the counts of enriched annotations were 10 for the protein domain families (Sonnhammer et al. 1997), two for Gene Ontology's cellular components (GO:CC), and two for the Gene Ontology's biological processes (GO:BP).

Principal component analyses (PCA) on identified proteins were performed by the ClustVis tool (Metsalu and Vilo 2015), which is coded in R. Using the *single* clustering method, a clustered heatmap of ALS condition‐exclusive protein expression patterns was generated with the R package *hclust*.

In silico interactome building was performed on the ALS‐associated proteins within the dataset using the software Ingenuity Pathway Analysis (IPA) (version 01‐22‐01, Qiagen, Hilden, Germany).

A global overrepresentation analysis (ORA) of the identified proteins, including proteins exclusively detected with significant confidence after Benjamini‐Hochberg correction (FDR < 0.01) in CAs from ALS brains, was performed with the Metascape online tool (Zhou et al. 2019) against several biological secondary databases, such as Gene Ontology (GO) and Reactome. The resulting network file was then processed with Cytoscape software version 3.10.2 (Shannon et al. 2003).

Semi‐quantification of proteins was performed by calculating the normalized spectral abundance factors (NSAFs) of proteins that were identified with a confidence of 95% (FDR < 0.05) and for which at least 10 peptides were measured, to filter out low‐abundance proteins and prevent false positives. NSAF has proven to be an effective quantification method for proteins based on their peptides (Zybailov et al. 2006). Differential expression analysis was performed with the limma package (version 3.64.1) in R. Simple significance thresholds were applied to maximize the information yield in this analysis (*p < 0.05*, Fold Change > 1.5). The heatmaps, volcano plot, PCA and ORA on these quantitative data were done in R version 4.5.1. ORA were conducted with the *gprofiler2* package version 0.2.3 in R (*p* < 0.05).

## 3 Results

### 3.1 Characterization of CAs Isolated From *Post‐mortem* Brain Tissues

**FIGURE 1:**
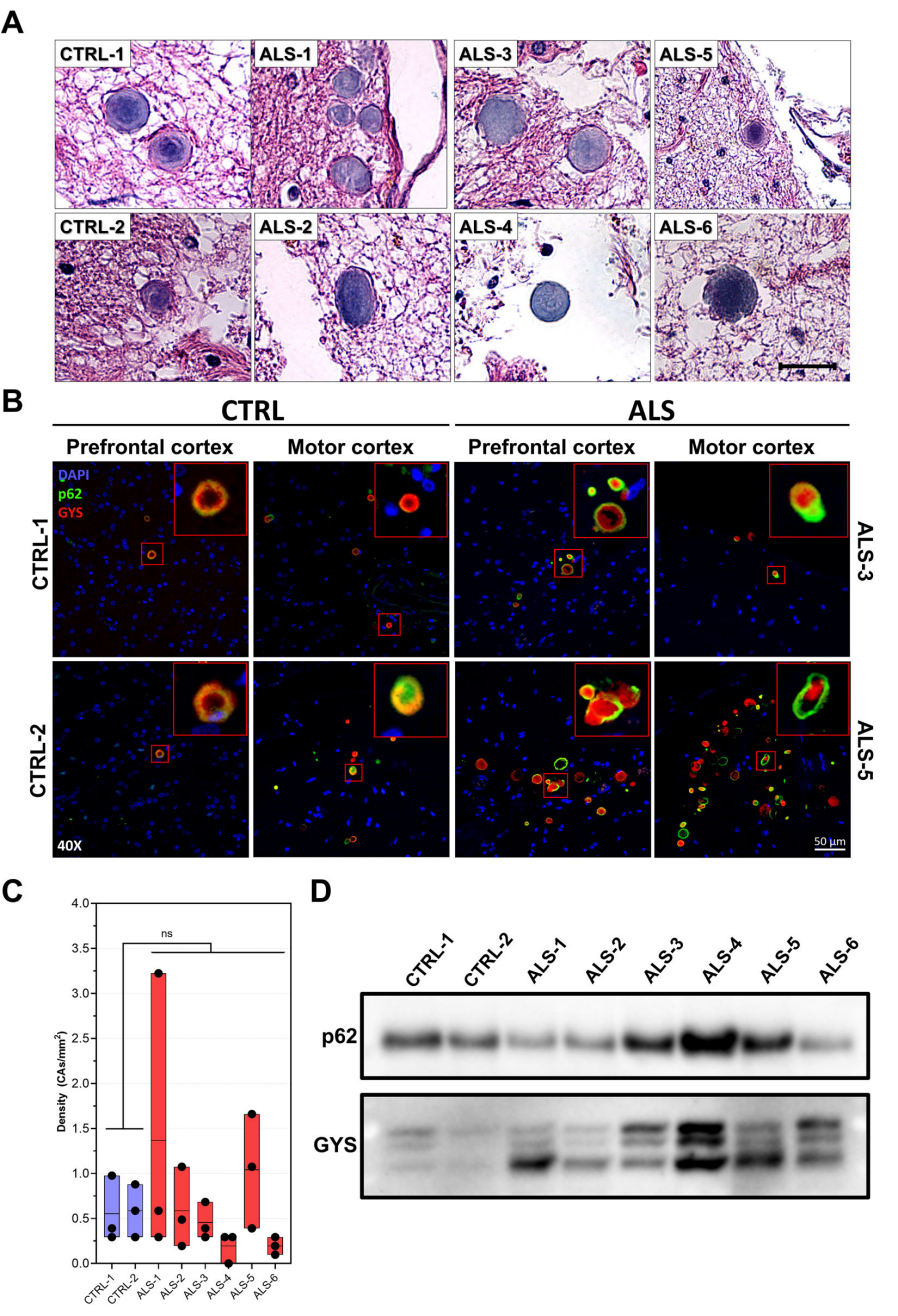
**Localization and extraction validation of CAs by immunofluorescence of CAs isolated from sporadic ALS brain tissues. (A)** Hematoxylin and eosin staining of CAs on 10 µm‐thick cross‐sections of the prefrontal cortex from the *post‐mortem* brains of six sALS patients and two non‐ALS controls. Scale bar = 20 µm. Magnification: 400X. **(B)** Indirect immunofluorescence carried on 10 µm‐thick brain sections from CTRL and sporadic ALS patients. Representative images showing the presence of p62 (green) and GYS (red) confirm the presence of CAs in the examined brain sections. **(C)** Barplot of CAs density in 10 µm‐thick parafilm‐embedded cross‐sections of the prefrontal cortex from the *post‐mortem* brains of sALS patients and non‐ALS controls. 3.2 mm x 3.2 mm (10.24 mm^2^) pictures were acquired. N_ALS_ = 6, N_CTRL_ = 2; n = 3. **(D)** Western blot of p62 and GYS expression in 10 µg of proteins from the CAs extracted from the prefrontal cortex.

The presence of CAs in different brain sections was confirmed both by bright field microscopy and immunofluorescence using CA‐specific markers. Hematoxylin‐ and eosin‐stained CAs were observable on 10 µm‐thick brain slices from every individual (Figure 1A). Immunofluorescence was done on 10 µm‐thick prefrontal and motor cortex sections using CA‐associated proteins, sequestosome‐1 (p62/SQSTM1) and glycogen synthase (GYS) commercial antibodies (Figure 1B). Although the number, density, and distribution of CAs seemed variable (Figure 1C), positively labeled CAs can be observed in both CTRL individuals and ALS patients, and no significant difference were noted between the two groups (Figure 1A and 1B). Proteins extracted from CAs were analysed by Western blot, and the expression of p62 and GYS was assessed to validate the extraction (Figure 1D).

### 3.2 ALS CAs Exhibit a Distinct Proteomic and Functional Landscape

**FIGURE 2:**
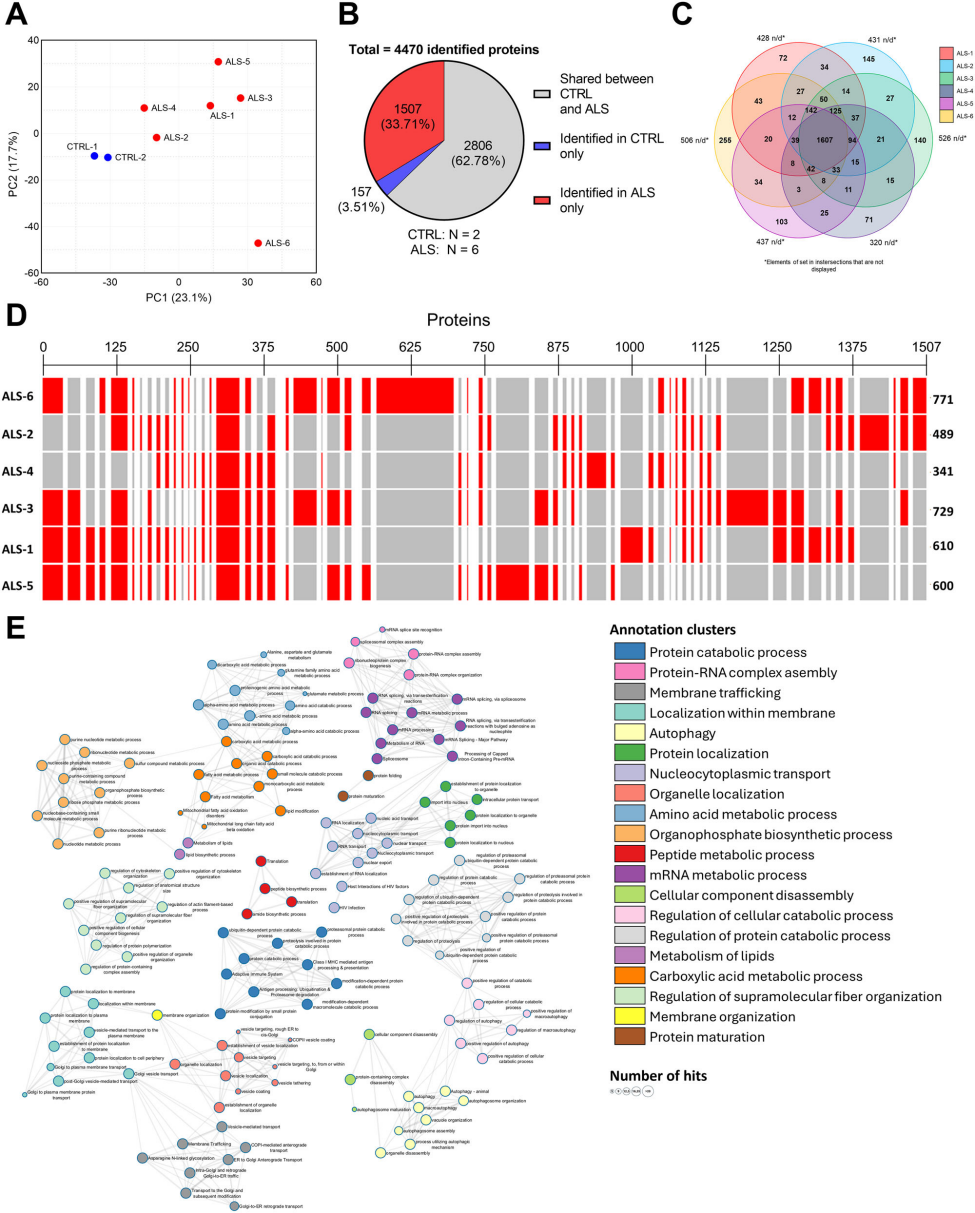
**Distribution of proteins identified in CAs extracted from the *post‐mortem* brain of all studied individual**. (**A**) PCA of proteins expression patterns among CAs from the eight patients. (**B**) Pie chart of proteins identified in CAs from both ALS and CTRL brains (62.78%), from ALS brains only (33.71%), or from CTRL brains only (3.51%) out of the 4470 identified proteins. (**C**) Venn diagram of proteins identified in CAs from ALS brains only, with a representation of the proteins that are shared between two or more ALS individuals. (**D**) Clustered heatmap of proteins identified in each ALS brain, clustered according to their individual distribution. (**E**) Protein sets interaction network of significantly enriched GO:BP, clustered according to ontology categories. Node color represents the ontology cluster and node size represents the number of hits the ORA returned. N_ALS_ = 6, N_CTRL_ = 2. *FDR* < 0.01.

We next conducted a comprehensive proteomic analysis to identify proteins detected in the isolated CAs. Proteomic profiling of CAs isolated from ALS and control brains revealed marked differences in protein content and functional enrichment. PCA showed a clear separation between ALS and control samples, indicating distinct global proteomic signatures (Figure 2A). Mass spectrometry identified 4470 proteins, with 1507 (33.71%) solely detected in the ALS group (N = 6), whilst only 157 (3.51%) were only detected in the CTRL group (Figure 2B). In contrast, 2806 proteins (62.78%) were common in both conditions.

A Venn diagram further demonstrated the distribution of CAs proteins across patients, highlighting both shared and unique protein subsets in each ALS individual, which may reflect the clinical heterogeneity characteristic of the disease (Figure 2C). Hierarchical clustering analysis revealed coherent groups of differentially enriched proteins, with ALS group‐exclusive proteins clustering underscoring the presence of ALS‐specific molecular signatures (Figure 2D).

To explore biological functions associated with these proteomic changes, annotation clustering and network analysis were performed using the Metascape online tool. The enrichment network revealed multiple interconnected annotation clusters, each corresponding to biological processes or pathways, including peptide catabolic and metabolic processes, which were strongly predominant, along with processes associated with lipid metabolism and autophagy (Figure 2E). Taken together, these results suggest that ALS CAs are characterized by a distinct proteomic identity and functional landscape compared to controls.

### 3.3 CAs Display Cellular Diversity and ALS‐exclusive Functional Profiles Identified by Enrichment Analysis

**FIGURE 3:**
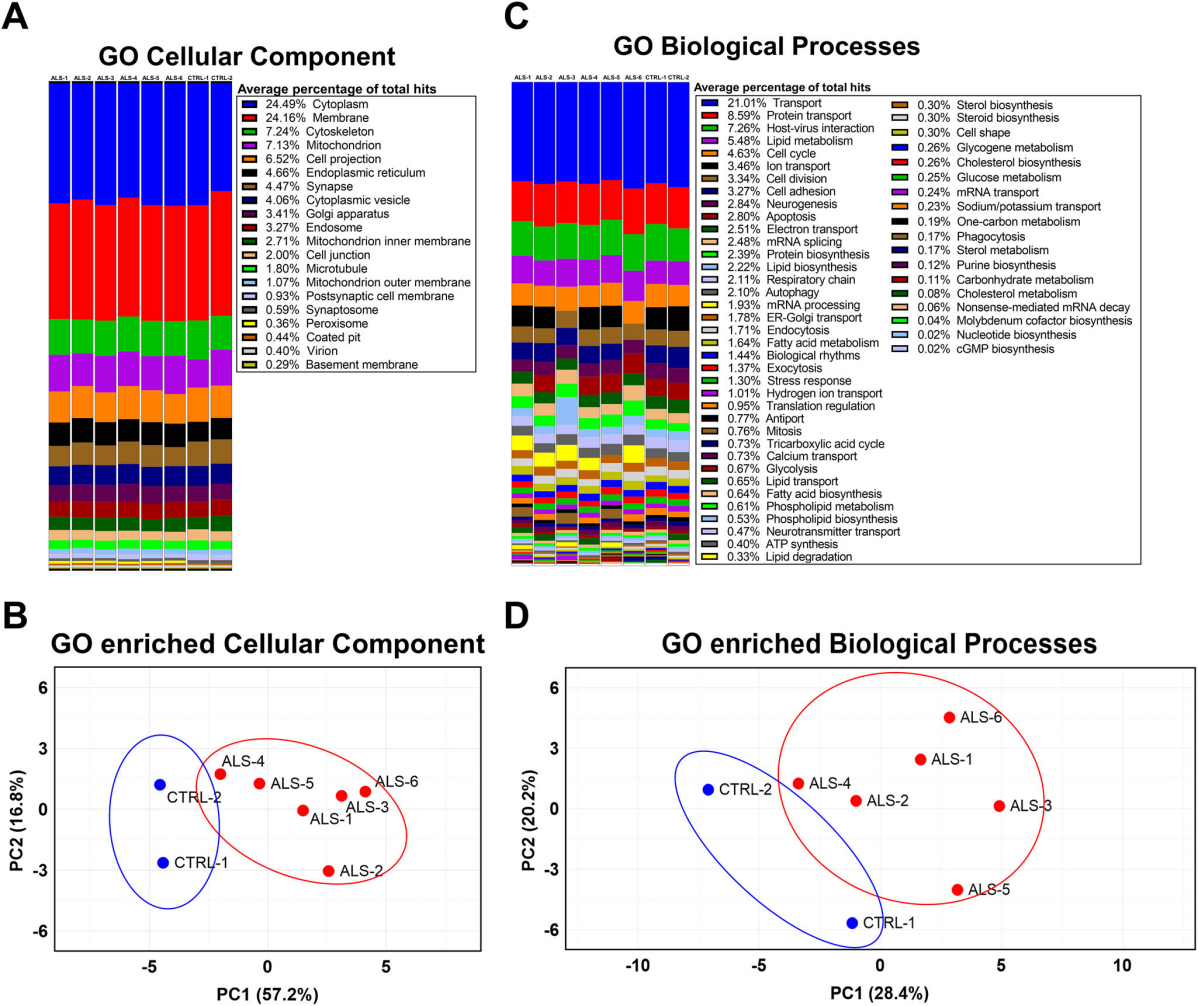
**Overview of the composition and cellular origin of proteins in CAs extracted from *post‐mortem* brains. (A)** Relative counts of significantly enriched GO:CC terms associated with proteins identified in CAs. **(B)** PCA of GO:CC annotation counts in CAs from CTRL and ALS individuals’ CAs **(C)** Relative counts of significantly enriched GO:BP associated with proteins identified in CAs. **(D)** PCA of GO:BP annotation counts in CAs from CTRL and ALS individuals’ CAs. N_ALS_ = 6, N_CTRL_ = 2.

The DAVID online bioinformatics tool was used to functionally annotate the protein list identified in CAs, thereby providing insight into their cellular compartment origin and functional composition. Twenty GO/cellular component terms with more than two hits were represented in the enrichment analysis (Figure 3A). The graphical representation across samples indicates that the cellular composition of CAs is relatively consistent among individuals (Figure 3A). Notably, a large fraction of proteins mapped to the cytoplasm (24.49%) and cell membrane (24.16%), while substantial proportions also originated from the cytoskeleton (7.24%) and mitochondria (7.13%), underscoring the multifaceted intracellular sources that contribute to CA's protein content (Figure 3A). Despite this seemingly heterogeneity in cell compartments categories, PCA computed from the number of hits per term still suggests distinct cluster between ALS CA and controls (Figure 3B). In contrast, greater variability was observed in the enrichment of GO:BP terms (Figure 3C‐D). 55 biological processes with more than two hits were represented, with the most prominent annotated terms including transport (21.01%), protein transport (8.59%), host‐virus interactions (7.26%), lipid metabolism (5.48%), cell cycle (4.63%), ion transport (3.46%), cell division (3.34%), cell adhesion (3.27%), neurogenesis (2.84%) and apoptosis (2.80%). These findings suggest that CAs integrate proteins from diverse cellular origins and pathways, potentially reflecting their role as accumulators of heterogeneous cellular material. Detailed counts for the enriched annotations are provided in the Supplementary Table 2.

### 3.4 In Silico Pathway Analysis Identified ALS‐related and Neurodegeneration‐linked Proteins

**FIGURE 4:**
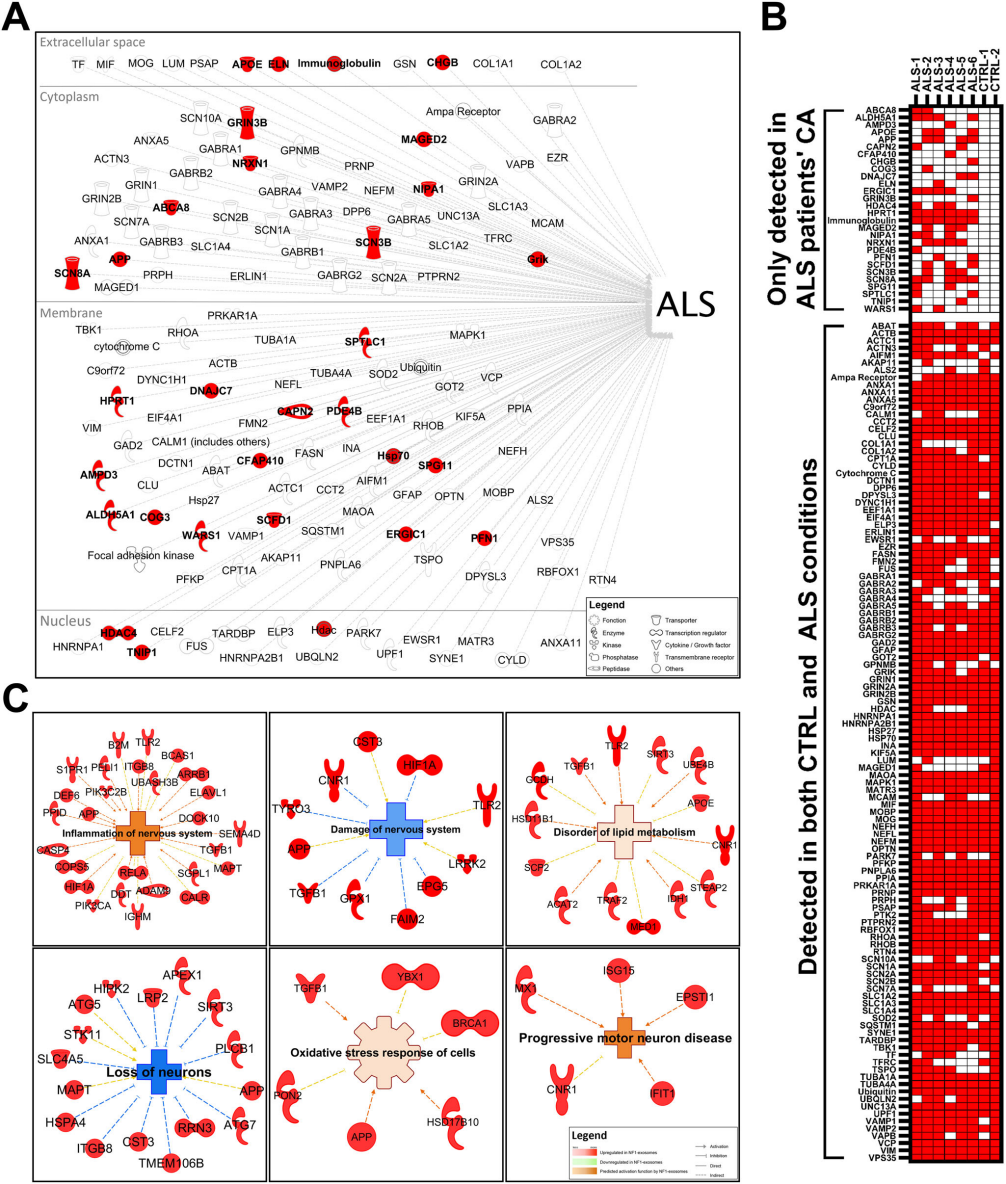
**Proteins associated with ALS and neurodegeneration‐related processes identified in CAs. (A)** In silico interactome of proteins associated with ALS detected CAs for both studied groups. Proteins highlighted in red are found exclusively in ALS patients’ CAs. **(B)** Heatmap presenting the ALS‐associated proteins (red) in the CAs among all ALS patients and control individuals. Proteins exclusive to the ALS condition are separated from others and displayed in the heatmap's top section. **(C)** Neurological disease‐associated mechanisms predicted as activated or inhibited by an overabundance of proteins exclusive to ALS CAs (*p* < 0.05). N_ALS_ = 6, N_CTRL_ = 2.

To gain deeper insight into disease‐related mechanisms and identify molecular pathways potentially contributing to neuronal loss in ALS, we performed in silico pathway analysis of the 4470 identified proteins extracted from isolated CAs using the IPA systematic literature query tool. This analysis revealed 142 proteins previously associated directly or indirectly with ALS (Figure 4A). Among these, 28 proteins were solely detected in CAs isolated from the brain ALS patients including ALD5H1, ERGIC1, HPRT1, Immunoglobulin, NRXN1 and SCN8A (Figure 4B). Other proteins such as TDP‐43, C9orf72, FUS, TBK1, NF‐L, UNC13A, VCP, VAPB, and ALS2 were detected in both ALS and controls, and showed heterogeneous detection across the six ALS patients (Figure 4B). Importantly, many of these proteins have established roles in neurodegeneration and are functionally linked to neuroinflammation, lipid metabolism disorder, damage of the nervous system, loss of neurons, progressive motor neuron disease and cellular stress, all of which are key elements of ALS progression (Figure 4C).

### 3.5 NSAF‐based Quantification Reveals a Proteomic Signature Specific to ALS Patients

**FIGURE 5:**
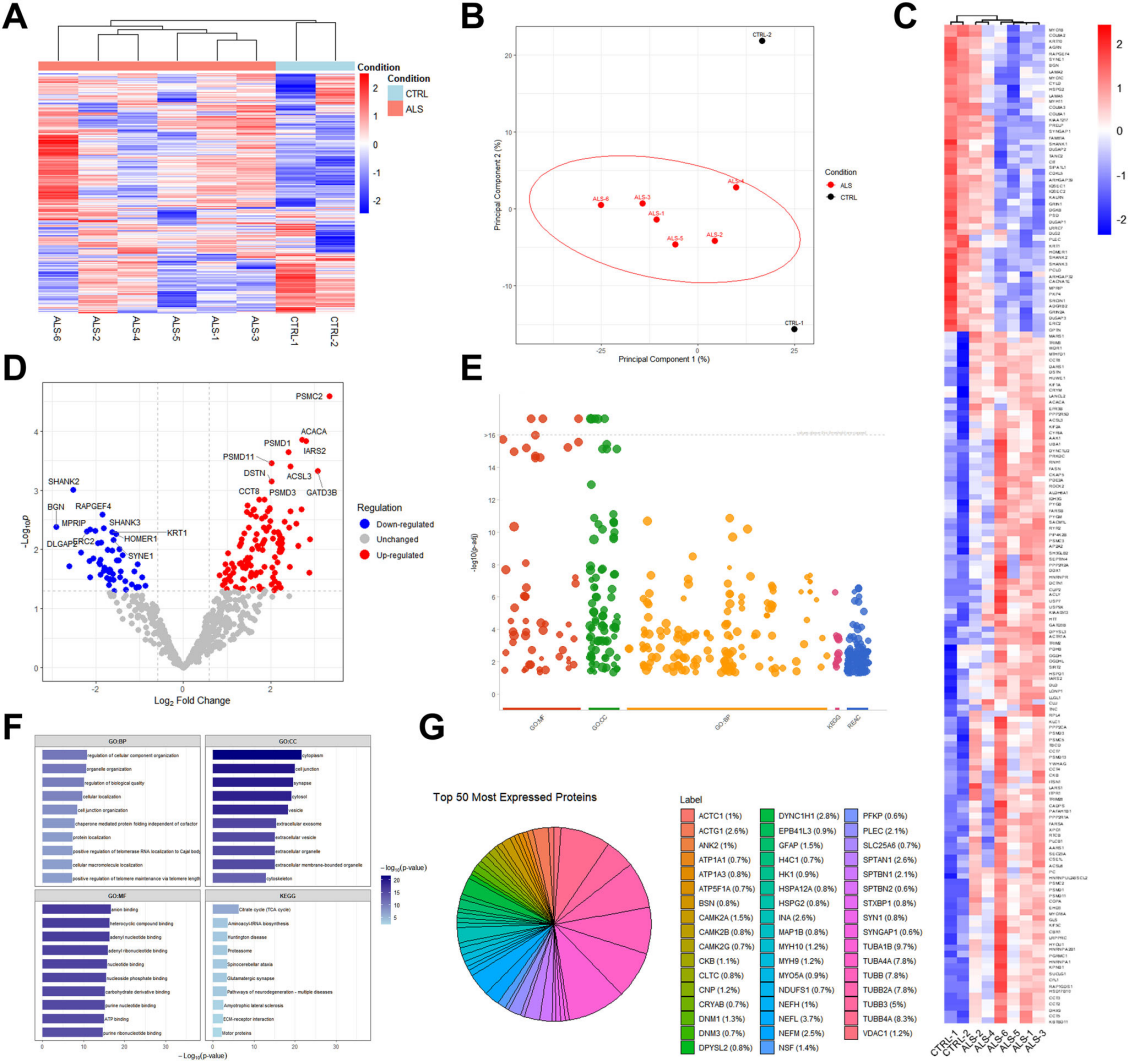
**NSAF estimation of protein counts in CAs from ALS patients and controls. (A)** Hierarchical clustering heatmap of 658 proteins qualifying for quantification using NSAF in CAs from ALS patients and CTRL. Protein expression levels are displayed as z‐scores. Dendrograms show sample clustering based on overall protein expression profiles. **(B)** PCA of the expression profiles in ALS samples and CTRL samples. The first two principal components are displayed, with percentage of variance explained indicated on each axis. **(C)** Hierarchical clustering heatmap focusing on the 166 significantly differentially expressed proteins between ALS and CTRL CAs. *P* < 0.05, Fold Change > 1.5. **(D)** Volcano plot displaying statistical significance (‐log_10_ *p*‐value, y‐axis) versus fold change (log_2_ fold change, x‐axis) for CAs proteins**. (E)** GO enrichment analysis results from g:Profiler showing significantly enriched biological processes and pathways among differentially expressed proteins. *P* < 0.05. **(F)** Barplots of GO:Biological Processes, GO:Cellular Compartments, GO:Molecular Functions and KEGG pathway significantly enriched in differentially expressed proteins in ALS CAs (*p* < 0.05). **(G)** Pie chart showing the relative abundance of the 50 most highly expressed proteins in CAs, with each segment representing individual proteins. N_CTRL_ = 2, N_ALS_ = 6.

To quantify protein abundance in CAs, we applied the NSAF method to the 658 proteins that met criteria for robust quantification across ALS patients and controls. Hierarchical clustering of these proteins revealed distinct expression patterns that clearly separated ALS from control samples, with coordinated up‐ and downregulated protein clusters (Figure 5A). PCA further confirmed this separation, as ALS samples grouped tightly together and were clearly distinguished from control samples along the first principal component, which explained the majority of variance in the dataset (Figure 5B). Differential expression analysis returned 166 proteins significantly altered in ALS CAs (*p* < 0.05, Fold Change ≥ 1.5) (Supplementary Table 3), further validating our first results (Figure 5C). Upregulated proteins included proteasome components (PSMC2, PSMD1, PSMD11, and PSMD3), metabolic enzymes (ACACA and ACSL3), and other proteins involved in cytoskeletal dynamics or protein quality control such as (IARS2, DSTN, GATD3B, and CCT8) (Figure 5D). Significantly downregulated proteins include synaptic scaffolding proteins SHANK2, SHANK3, and HOMER1, extracellular matrix component BGN, cytoskeletal proteins KRT1 and SYNE1, and signaling proteins RAPGEF4, MPRIP, DLGAP5, and PRC2 (Figure 5D).

GO enrichment analysis of the differentially abundant proteins highlighted pathways related to protein folding, stress response, metabolic processes, and protein quality control (Figure 5E‐5F). The 50 most highly expressed proteins are mostly related to cytoskeletal components, particularly tubulin family proteins including α‐tubulins (TUBA1B, TUBA4A) and β‐tubulins (TUBB3, TUBB, TUBB2A, and TUBB4A). Neurofilament proteins are also highly represented, including NEFL and NEFM, along with cytoskeletal organizing proteins, such as spectrins (SPTAN1, and SPBTBN1), actin gamma 1 (ACTG1) and plectin (PLEC), and diverse metabolic enzymes, chaperones, and signaling proteins (Figure 5G).

## 4 Discussion

In this study, we provide the first comprehensive proteomic characterization of CA isolated from *post‐mortem* brain tissue of ALS patients compared with controls. By combining biochemical extraction, validation through Western blotting, NSAF‐based quantitative proteomics, enrichment analysis, and pathway annotation, we demonstrate that CAs harbour distinct protein signatures reflective of ALS pathology. Our findings establish CAs not merely as inert byproducts of neurodegeneration but as potential reservoirs of disease‐relevant proteins, with implications for biomarker discovery and mechanistic insight. The prefrontal cortex was selected because of its relevance to the ALS‐FTD clinicopathological spectrum, where frontal involvement and shared alterations in protein homeostasis are well documented. In addition, previous studies have also reported abundant CA accumulation in the frontal and temporal cortices (Alsina et al. 2024, Cavanagh 1999).

The success of CAs extraction from the brains has been previously validated in Pisa et al. 2018 (Pisa et al. 2018) by assessing the expression of p62 and GYS, which has again been confirmed in this study. In forthcoming investigations aimed at quantifying CAs across various brain regions in different patient samples, the simultaneous detection of these markers via immunofluorescence could enable the evaluation of the number of CAs in a given brain. While these markers support the efficiency of CA enrichment, consideration of the potential co‐isolation of other tissue components is important for accurate interpretation of downstream proteomic data. Pisa et al. 2018 reported that the enriched CA fraction lacks significant cellular and mitochondrial markers and concluded that the collected pellet is largely composed of CAs (Pisa et al. 2018). Although the presence of trace amounts of residual cellular or organelle‐derived material cannot be entirely excluded, such components may reflect debris naturally associated with CAs rather than technical contamination.

### 4.1 CAs as Reservoirs of Dysfunctional Proteins, and Cellular Origin

The enrichment of diverse proteins from multiple cellular compartments, including cytoplasm, plasma membrane, cytoskeleton, and mitochondria, supports the concept that CAs accumulate materials derived from widespread neuronal and glial stress. This is consistent with prior observations that CAs act as cellular waste repositories (Selmaj et al. 2008, Wilhelmus et al. 2011). Importantly, our data show that ALS CAs contain proteins implicated in maintaining cellular homeostasis, metabolic function, cytoskeletal remodeling, and proteostasis, further supporting the view that ALS CAs serve as reservoirs for dysfunctional proteins.

### 4.2 Distinctive ALS Proteomic Signatures

Our enrichment and clustering analyses revealed coherent separation of ALS from control CAs, indicating the presence of protein profiles specific to the disease group. Hierarchical clustering identified groups of differentially enriched proteins that robustly segregated ALS samples, while functional annotation emphasized pathways related to stress responses, metabolism, neuroinflammation, and synaptic regulation. This supports the notion that ALS CAs retain disease‐related molecular imprints, which may serve as signatures of neurodegenerative pathology.

While protein identification provided a qualitative overview of CAs’ composition, NSAFs calculation for each protein provided a supportive quantitative assessment of their protein content (Zybailov et al. 2006). Upregulation of proteasome components (PSMC2, PSMD1, PSMD11 and PSMD3), along with CCT8 and VCP‐related pathways) suggests an activated, yet likely insufficient, proteostasis response to protein misfolding. Enrichment of metabolic enzymes indicate altered lipid metabolism. In contrast, reduced synaptic and signaling proteins point to synaptic dysfunction and impaired cellular interactions. The predominance of cytoskeletal proteins, including tubulins (TUBA4A) and neurofilaments (NF‐L, NF‐M), along with spectrins and plectin, highlights cytoskeletal disruption and axonal damage. The presence of TDP‐43‐, FUS‐ and UNC13A‐related signatures further links CAs to altered RNA metabolism and synaptic dysfunction. Additionally, proteins such as TBK1 and C9orf72 implicate autophagy and neuroimmune pathways in CA composition. Altogether, these findings indicate that CAs integrate multiple converging mechanisms of ALS pathology including protein clearance deficits, cytoskeletal fragmentation, cellular stress (Pisa et al. 2018, Augé et al. 2018, Kumar et al. 2016). Consistent with proposed models of CA biogenesis, alterations in extracellular matrix‐related proteins reflect the extracellular remodeling necessary for their extrusion. Overall, these findings suggest that accumulation of proteins from multiple pathways within CAs reflects a complex cellular pathological response in ALS, which may function both as protective clearance sinks and as reservoirs of pathological protein aggregation.

Moreover, inter‐individual variability in the representation of certain ALS‐related proteins (TDP‐43, C9orf72, VCP and FUS) highlights the molecular heterogeneity of ALS, paralleling its well‐recognized clinical heterogeneity. For instance, TDP‐43 forms cytoplasmic inclusions in ∼95% of sALS cases (Arseni et al. 2022), disrupting RNA processing, while only 1.5% of sALS patients carry a mutation in the *TARDBP* gene (Mackenzie et al. 2010, de Boer et al. 2021). C9orf72 protein levels are also reduced in some sporadic cases, impairing autophagy. VCP dysfunction through oxidative damage contributes to protein aggregation and failure of quality control (Vanderhaeghe et al. 2024). On the other hand, FUS shows cytoplasmic mislocalization in about 1% of sporadic cases, disrupting RNA metabolism (Yang et al. 2025). Additionally, alsin (ALS2) loss‐of‐function affects endosomal trafficking and motor neuron survival, though its role in sporadic ALS is less established (Hadano et al. 2016). In this context, future studies could also help uncover underexplored pathological mechanisms in sALS. Furthermore, the detection of multiple ALS‐associated proteins and pathways signature within CAs reflects disease heterogeneity, with potential for patient stratification and biomarker development. Further investigation of CA biogenesis and protein sequestration will clarify whether these structures exert protective or pathogenic roles, as reservoirs of dysfunctional proteins in ALS.

### 4.3 Utility of MAiD‐derived *Post‐mortem* Tissue

An emerging opportunity for ALS research lies in the use of *post‐mortem* tissue collected from patients who receive MAiD. Compared to conventional end‐stage *post‐mortem* tissue, which often reflects profound neurodegeneration with near‐complete loss of motor neurons, MAiD‐derived samples may capture a disease state in which motor neurons and associated networks are still partially preserved. This provides a valuable window into earlier or intermediate stages of ALS pathology, complementing the advanced degenerative changes typically studied in end‐stage tissue. For proteomic analyses of CAs, this approach may reveal disease‐relevant proteins and pathways that are otherwise obscured at terminal stages, thereby enhancing mechanistic insight and improving stratification. Recent reports have described the establishment of MAiD‐related brain donation programs in ALS and other neurodegenerative diseases, highlighting both their scientific value and the need for careful ethical oversight to ensure respect for patient autonomy and informed consent (Downar et al. 2019). These initiatives underscore the potential of MAiD‐derived tissue to complement traditional *post‐mortem* studies while maintaining the highest ethical standards.

### 4.4 Limitations and Perspectives

Our study is limited by the relatively small sample size inherent to *post‐mortem* human tissue research and by inter‐individual variability in CA protein content. While NSAF‐based quantification provides robust relative abundance estimates, complementary intensity‐based or targeted proteomics could refine absolute quantification of disease‐relevant proteins. Finally, optimized methods to detect phosphorylated or misfolded protein species could also refine the quantification of disease‐relevant proteins.

Another limitation is the use of pathological non‐control samples rather than neurologically healthy donors, reflecting the limited availability of healthy *post‐mortem* brain tissue. As a result, underlying cellular and molecular alterations associated with ARSACS and PACNS may influence the CA proteomic composition, potentially attenuating the differences between ALS and control samples. Accordingly, the primary objective of the study was to characterize the proteomic composition of CAs, a structure whose molecular content remains poorly defined, rather than to draw definitive conclusions regarding ALS‐specific molecular features. The marked heterogeneity observed in CA protein composition further supports this descriptive focus. Age is another factor to consider, as CA accumulation is associated with brain aging (Riba et al. 2022, Pirici and Margaritescu 2014). In this study, control donors were younger than ALS donors, reflecting an age mismatch between groups. Because CA composition may evolve with aging, age‐related effects cannot be excluded as potential contributors. Future studies including age‐matched healthy donors will be essential to fully delineate disease‐specific signatures, and findings should therefore be interpreted with caution.

Extending analyses to the spatial dimension of CAs may enable quantitative assessments of their regional abundance and composition, as well as to reveal associations with local metabolic activity and neurodegenerative processes (Riba et al. 2021, Cavanagh 1999). Inter‐individual variability in CA abundance and composition may further relate to differences in disease progression and molecular pathology (Auge et al. 2017).

By sequestering stress‐ and clearance‐related proteins, CAs may provide insights into molecular processes activated in the diseased CNS. Future work should validate these findings in larger cohorts and assess whether CA‐derived proteins, potentially released into cerebrospinal fluid or peripheral compartments, can be detected in biofluids. Their heterogeneous proteomic composition further suggests potential utility for exploring inter‐individual variability in ALS, supporting their consideration as an underexplored source of disease biomarkers.

## 5 Conclusions

This study represents a significant step in understanding the composition and potential function of CAs in ALS. Altogether, this work provides a comprehensive proteomic map of ALS CAs and demonstrates that they are enriched in disease‐associated proteins involved in relevant pathogenetic pathways. Our findings reveal a distinct proteomic signature in ALS patients‐derived CAs, positioning them as promising reservoirs for biomarker discovery and offering new insight into ALS pathophysiology.

## Author Contributions

**Alexandre Paquet**: formal analysis, methodology, writing – original draft, writing – review and editing. **Lydia Touzel‐Deschênes**: conceptualization, formal analysis, methodology, writing – original draft, writing – review and editing. **Vincent Roy**: writing – original draft, writing – review and editing. **Stephan Saikali**: writing – review and editing. **Nicolas Dupré**: funding acquisition, supervision, writing – review and editing. **François Gros‐Louis**: conceptualization, formal analysis, funding acquisition, supervision, writing – review and editing.

## Conflicts of Interest

The authors declare no conflict of interest.

## Funding

F.G.‐L. is the recipient of a tier‐1 Canada Research Chair. A.P. is the recipient of a scholarship from the CHU de Québec Foundation—Desjardins. This project has been funded in part by the Canadian Institutes of Health Research (CIHR) and the Canada Foundation for Innovation (CFI).

## Institutional Review Board Statement

Not applicable.

## Informed Consent Statement

Informed consent was obtained from all participants who participated in the Canadian MAiD program.

## Language editing statement

The authors used ChatGPT (OpenAI, San Francisco, CA, USA) for assistance with English language editing. All scientific content, data interpretation, and conclusions were conceived, written, and verified solely by the authors.

## Supporting information




**Supplementary Table 1: Description of the patients whose *post‐mortem* brain tissues were collected for the study**. sALS = sporadic amyotrophic lateral sclerosis, ARSACS = autosomal recessive spastic ataxia of Charlevoix‐Saguenay, PACNS = primary angiitis of the central nervous system.

**Supplementary** **Table 2**: **Counts of GO:Cellular Components and GO:Biological Processes highlighted by ORA done on proteins identified in CAs**. *p*‐value < 0.05)

**Supplementary** **Table 3**: Summary of the 166 differentially expressed proteins in ALS CAs from the *limma* **differential** **expression analysis done on the** **NSAFs**. *p*‐value < 0.05, FC > 1.5. N_ALS_ = 6, N_CTRL_ = 2.

## Data Availability

Data available on request from the authors.
