# Supplementary material for: Proteomic Analysis of Corpora Amylacea Extracted From Post‐mortem Brain of MAiD‐end‐of‐life Sporadic ALS Patients
Source: Brain Behav. 2026 May 24;16(5):e71486. doi: 10.1002/brb3.71486 (PMC13238840; doi:10.1002/brb3.71486)
Supplement: Supplementary file 1 — Supplementary Table 1: Description of the patients whose post‐mortem brain tissues were collected for the study. sALS = sporadic amyotrophic lateral sclerosis, ARSACS = autosomal recessive spastic ataxia of Charlevoix‐Saguenay, PACNS = primary angiitis of the central nervous system. [file BRB3-16-e71486-s003.docx]

| Patient | Sex | Age at death (years) | | Condition |
| --- | --- | --- | --- | --- |
| ALS-1 | Male | 68 | sALS | |
| ALS-2 | Male | 70 | sALS | |
| ALS-3 | Female | 71 | sALS | |
| ALS-4 | Male | 71 | sALS | |
| ALS-5 | Male | 81 | sALS | |
| ALS-6 | Male | 81 | sALS | |
| CTRL-1 | Male | 38 | ARSACS | |
| CTRL-2 | Male | 56 | PACNS | |

**Supplementary Table 1. Description of the post-mortem brain tissue donors.** sALS = sporadic amyotrophic lateral sclerosis, ARSACS = autosomal recessive spastic ataxia of Charlevoix-Saguenay, PACNS = primary angiitis of the central nervous system.
