# Supplementary material for: Proteomic Analysis of Corpora Amylacea Extracted From Post‐mortem Brain of MAiD‐end‐of‐life Sporadic ALS Patients
Source: Brain Behav. 2026 May 24;16(5):e71486. doi: 10.1002/brb3.71486 (PMC13238840; doi:10.1002/brb3.71486)
Supplement: Supplementary file 2 — Supplementary Table 2: Counts of GO:Cellular Components and GO:Biological Processes highlighted by ORA done on proteins identified in CAs. p‐value < 0.05) [file BRB3-16-e71486-s002.docx]

**Supplementary Table 2. Counts of GO:Cellular Components and GO:Biological Processes highlighted by ORA done on proteins identified in CAs.** *P*-value < 0.05)
