# Supplementary material for: Proteomic Analysis of Corpora Amylacea Extracted From Post‐mortem Brain of MAiD‐end‐of‐life Sporadic ALS Patients
Source: Brain Behav. 2026 May 24;16(5):e71486. doi: 10.1002/brb3.71486 (PMC13238840; doi:10.1002/brb3.71486)
Supplement: Supplementary file 3 — Supplementary Table 3: Summary of the 166 differentially expressed proteins in ALS CAs from the limma differential expression analysis done on the NSAFs. p‐value < 0.05, FC > 1.5. NALS = 6, NCTRL = 2. [file BRB3-16-e71486-s001.docx]

**Supplementary Table 3: Summary of the 166 differentially expressed proteins in ALS CAs from the *limma* differential expression analysis done on the NSAFs.** *P*-value < 0.05, FC > 1.5. N_ALS_ = 6, N_CTRL_ = 2.
